# The Unripe Carob Extract (*Ceratonia siliqua* L.) as a Potential Therapeutic Strategy to Fight Oxaliplatin-Induced Neuropathy

**DOI:** 10.3390/nu17010121

**Published:** 2024-12-30

**Authors:** Laura Micheli, Marilena Muraglia, Filomena Corbo, Daniel Venturi, Maria Lisa Clodoveo, Roberta Tardugno, Valentina Santoro, Anna Lisa Piccinelli, Lorenzo Di Cesare Mannelli, Stefania Nobili, Carla Ghelardini

**Affiliations:** 1 Department of Neurosciences, Psychology, Drug Research and Child Health (NEUROFARBA), Section of Pharmacology and Toxicology, University of Florence, 50139 Florence, Italy; daniel.venturi1@unifi.it (D.V.); lorenzo.mannelli@unifi.it (L.D.C.M.); stefania.nobili@unifi.it (S.N.); carla.ghelardini@unifi.it (C.G.); 2 Department of Pharmacy-Drug Sciences, University of Bari Aldo Moro, 70125 Bari, Italy; filomena.corbo@uniba.it (F.C.); roberta.tardugno@uniba.it (R.T.); 3 Interdisciplinary Department of Medicine, School of Medicine, University of Bari Aldo Moro, 70124 Bari, Italy; marialisa.clodoveo@uniba.it; 4 Department of Pharmacy, University of Salerno, 84084 Fisciano, Italy; vsantoro@unisa.it (V.S.); apiccinelli@unisa.it (A.L.P.); 5 National Biodiversity Future Center (NBFC), 90133 Palermo, Italy

**Keywords:** oxaliplatin, neurotoxicity, pain, *Ceratonia siliqua* L., unripe carob pods, polyphenols

## Abstract

Background: Oxaliplatin-induced neuropathy (OIN) is a severe painful condition that strongly affects the patient’s quality of life and cannot be counteracted by the available drugs or adjuvants. Thus, several efforts are devoted to discovering substances that can revert or reduce OIN, including natural compounds. The carob tree, *Ceratonia siliqua* L., possesses several beneficial properties. However, its antalgic properties have not been substantially investigated and only a few investigations have been conducted on the unripe carob (up-CS) pods. Thus, the aims of this study were to evaluate for the first time the unripe variety of Apulian carob, chemically characterized and profiled as antioxidant potential of polyphenolic compounds as well as to investigate the ability of up-CS to reduce the neurotoxicity in a mouse model of oxaliplatin-induced neuropathic pain. Methods: By UHPLC-HRMS/MS analyses, 50 phenolic compounds, belonging mainly to n-galloylated glucoses and flavonoids were detected. Results: In a mouse model of oxaliplatin-induced neurotoxicity (2.4 mg/kg, 10 injections over two weeks), acute *per os* treatment with up-CS provoked a dose-dependent pain-relieving effect that completely counteracted oxaliplatin hypersensitivity at the dose of 200 mg/kg. Repeated oral administration of up-CS (100 mg/kg), concomitantly with oxaliplatin injection, exerted a protective effect against the development of thermal and mechanical allodynia. In addition, up-CS exerted a neuroprotective role against oxaliplatin-induced astrocytes activation in the spinal cord measured as GFAP-fluorescence intensity. Conclusions: Overall, our study contributes to the knowledge on up-CS properties by highlighting its protective activity in the painful condition related to the administration of oxaliplatin.

## 1. Introduction

Neuropathic pain is defined by the International Association for the Study of Pain (IASP) as being caused directly by damage or illness affecting the somatosensory system [1]. Several painful pathological conditions, for instance, postherpetic neuralgia, diabetic or HIV-related neuropathy, and cancer-related pain, can influence the patient’s quality of life significantly [2]. However, chronic peripheral neuropathy may also develop following the administration of neurotoxic anticancer agents such as coordination complexes of platinum, taxanes, Vinca alkaloids, bortezomib as well as immune checkpoint inhibitors and others [3,4]. Among these drugs, one of the most used is oxaliplatin, a platinum compound that is mainly administered to all patients affected by gastrointestinal cancers. An important aspect is that while a relevant percentage of patients treated with oxaliplatin-based chemotherapy, especially those with early-stage cancers, will be cured with a life expectancy like that of health subjects, they will continue to suffer from the oxaliplatin-induced peripheral neuropathy (OIN) [5].

To date, no drugs or adjuvants able to revert and/or contribute to ameliorating this anticancer drug side effect are available [6]. Off-label antidepressants, anticonvulsants, and opioids are commonly used, even in combination, to reduce this form of peripheral neuropathy. However, these pharmacological treatments are expected to induce their specific side effects (e.g., tolerance in the case of opioids). Thus, preclinical and clinical research is strongly committed to discovering substances that can safely revert or reduce the OIPN. Several, of the mostly investigated substances to this aim, derive from natural sources, mainly from the plant kingdom, being this strategy supported by the wide variety of available compounds [7].

The carob tree, *Ceratonia siliqua* L., is a Mediterranean perennial evergreen tree belonging to the Fabaceae (Leguminosae) family [8,9].

Several medicinal properties of *C. siliqua* have been described, mainly related, but not limited to, gastrointestinal disorders and diabetes. However, a field that has not been substantially explored is represented by the potential use of *C. siliqua* in improving pain syndromes. A recent paper investigated the anti-nociceptive and anti-inflammatory properties of *C. siliqua* leaves ethanol extracts in mice, suggesting a potential correlation between the reduced nociception and the presence of specific substances, in particular myricetin [10].

It is, in fact, well known that *C. siliqua* contains several potentially beneficial compounds including polyphenols (e.g., tannic acid, gallic acid), flavonoids (e.g., quercetin, myricetin), carbohydrates, minerals, and proteins [9,11].

However, while much scientific evidence is available on the bioactive properties of ripe carob, few investigations have been conducted on the unripe (or green) carob. Differences between ripe and unripe carobs have been shown for instance in terms of the total soluble solids, maturity index, and sugar content that have been found to be increased in the ripe carob compared with the unripe carob or in terms of titratable acidity, protein, total phenolic contents, and antioxidant activity that have been found to be decreased in the ripe carob compared with the unripe one [12]. The gallotannin amount has been found, for instance, to be higher in the extract of the unripe carob pulp compared with the ripe one [12] and, more in general, the available studies agree on a higher content in gallic acid and related derivatives as well as in tannins in the unripe carob pulp compared with the ripe carob pulp [11,12,13].

As observed for the ripe carob pods, differences in the physicochemical and phytochemical composition of the unripe carob pods may also exist either according to the location of carob collection, as recently reported for wild unripe carobs grown up in three different places in Algeria [14] or according to carob varieties (e.g., Wild, Sisam, and Fleshy) [12]. The unripe Sisam variety showed, in fact, the highest amount of total phenolics whereas the ripe Wild variety showed the lowest amount [12,13].

The optimization of the processes aimed at extracting bioactive compounds from the complex matrix of carob pods play an important role in the framework of potential phytotherapeutic and/or drug adjuvant uses.

Traditional methods are represented by maceration and heating-assisted extraction. More innovative methods are represented by ultrasonic-assisted extraction (UAE) [15]. Recently, we also contributed to improve the extraction technique of polyphenols from ripe carob pods by optimizing a screening Plackett-Burman design and non-standard central composite design coupled to response surface methodology integrated with statistical tools [16,17].

Overall, the aims of this study were to evaluate for the first time the unripe variety of Apulian carob pods, chemically characterized and profiled as antioxidant potential of polyphenolic compounds as well as to investigate the ability of the above-mentioned unripe carob to reduce the neuropathic pain in a mouse model of oxaliplatin-induced neurotoxicity.

## 2. Materials and Methods

### 2.1. Sampling

In a plantation on a private property called Masseria Agricola Olere, located in the Apulia region of southern Italy, in Ostuni (BR) (40°46′06″ N 17°32′52″ E, altitude above sea level: 54 m), two different cultivars of the *Ceratonia siliqua* tree, wild (named Selvatica) and Amele were produced under identical agronomic and environmental conditions. *C. siliqua* unripe pods samples used in this study came from this area.

*C. siliqua* spreads through seeds. The wild variety (Selvatica) does not possess the important productive characteristics of the plant from which the fruit comes; it develops from seeds and is of little agronomic importance. The young plants are then grafted with strains of suitable varieties. The most popular cultivar for grafting in Puglia is Amele.

Unripe pods were harvested from mature trees (more than 100 years old), starting outside and working in the same way in each tree.

Disease-free samples were collected in May 2022, according to the specific sampling time. A voucher specimen of the collected fruit samples has been deposited at the University of Bari Aldo Moro, Department of Pharmacy-Drug Sciences, under the acquisition number up-CS.

### 2.2. Ceratonia siliqua L. Pods Extract (up-CS) Preparation

The whole unripe pods of *C. siliqua* (pulp and seeds) were washed and stored in a refrigerator at −80 °C before being freeze-dried (Christ Alpha 1-4 LSC9) and ground with a blade mill, obtaining, by sieving, a powder whose particles had a diameter of 200 mm.

The powder (6 g) was extracted in an ultrasonic bath (500 W as power and 37 KHz as frequency) using H_2_O (200 mL) as solvent for 30 min, at a temperature of 25 °C. The extract obtained was filtered and freeze-dried again.

### 2.3. Chemicals and Standards

MS-grade acetonitrile, water and formic acid were obtained from Romil (Deltek, Italy). Reference standards of natural compounds were provided by Extrasynthese (Lyon, France) or Merck Chemicals (Milan, Italy).

### 2.4. UHPLC-HRMS/MS Analyses

A Vanquish Flex UHPLC system interfaced to Diode Array Detector FG and Orbitap Exploris 120 mass spectrometer (ThermoFisher Scientific, Milano, Italy) was employed for untargeted analysis of unripe carob pod extract up-CS. Chromatographic separation was performed using a Kinetex C18 column (2.1 mm × 100 mm, 2.6 μm; Phenomenex, Bologna, Italy), protected by a C18 Guard Cartridge (2.1 mm I.D.), and H_2_O (solvent A) and acetonitrile (solvent B), both acidified by 0.1% formic acid *v*/*v*, with the following binary gradient: 0–3 min at 2% of B, 3–5 min 2–8% B; 5–9 min at 8% B, 9–16 min 8–18% B, 16–21 min 18–30% B, 21–25 min 30–98% B. The flow rate was set to 0.5 μL min^−1^ and the column oven was set to 40 °C. Mass spectrometer was operated in negative ionization mode using a Full MS data-dependent MS/MS acquisition mode with a stepped collision energy HCD (20, 40, and 60). The resolution of Full MS (range 100–1000 *m*/*z*) and dd-MS2 scans were set at 30 k (FWHM). Xcalibur software (Version 4.4, ThermoFisher Scientific) was used for instrument control, data acquisition and data analysis. UV spectra were acquired in the 200–600 nm range.

Detected compounds were characterized based on negative HRMS/MS data, retention times and UV spectra. Identification level was established according to metabolomics standards initiative [18]. Unambiguous identifications (level 1) were achieved by comparison with reference standards analyzed under the same experimental conditions. Putative identification (level 2) was based on MS data similarity with literature data or public databases and further supported by chemotaxonomic data. When no literature/database data are available for proposed structures, an identification level 3 was established by spectral similarity to chemical class of compounds and chemotaxonomic data.

### 2.5. Animals

For all the experiments described below, male CD-1 mice (Envigo, Varese, Italy) weighing approximately 20 to 25 g at the beginning of the experimental procedure were used. Animals were housed in Ce.S.A.L. (Centro Stabulazione Animali da Laboratorio, University of Florence, Florence, Italy) and used at least 1 week after their delivery. Eight mice were housed per cage (size 26 × 41 cm^2^); animals were fed *ad libitum* with standard laboratory diet and tap water, kept at 23 ± 1 °C with a 12-h light–dark cycle, light at 7 a.m.

### 2.6. Oxaliplatin-Induced Neuropathy and Treatment with up-CS

Oxaliplatin 2.4 mg/kg was dissolved in 5% glucose solution and administered intraperitoneally (i.p.) for 5 consecutive days every week for 2 weeks (10 i.p. injections) [19,20]. Control animals received an equivalent volume of 5% glucose i.p. (vehicle).

For the evaluation of the therapeutic effect (acute experiment), up-CS was suspended in 1% carboxymethylcellulose sodium salt (CMC; Sigma Aldrich, Milan) and *per os* administered at the doses of 30, 100 and 200 mg/kg when neuropathy was well established (day 15). For the evaluation of the protective effect (repeated treatment), up-CS 100 mg/kg was suspended in 1% CMC and *per os* administered for 5 consecutive days every week for 2 weeks (10 treatments) starting from the same day of oxaliplatin injection.

### 2.7. Assessment of Mechanical Allodynia, Von Frey Test

The response of the mouse against a mechanical non-noxious stimulus was measured using the von Frey test. Habituation of 15 min was allowed before the test after placing the animals in 20 × 20-cm plexiglass boxes equipped with a metallic meshy floor, 20 cm above the bench. An electronic von Frey hair unit (Ugo Basile, Varese, Italy) was used: the withdrawal threshold was measured by applying a force ranging from 0 to 5.0 g with a 0.2-g accuracy. A punctuate stimulus was directed at the midplantar area of each anterior paw from below the meshy floor with a plastic tip and the withdrawal threshold was automatically displayed on the screen. The paw pain threshold was outlined as the minimum pressure required to obtain an immediate and robust withdrawal reflex of the paw. Voluntary movements associated with locomotion were not taken as a withdrawal response. Stimuli were applied on each posterior and repeated 5 times. The final value was obtained by averaging the 5 measures [21].

### 2.8. Assessment of Thermal Allodynia, Cold Plate Test

The response of the mouse against a thermal non-noxious stimulus was assessed using the Cold plate test. With minimal animal–handler interaction, animals were placed onto the surface of the Cold plate (Ugo Basile, Varese, Italy) maintained at a constant temperature of 4 ± 1 °C. Ambulation was restricted by A cylindrical plexiglass chamber (diameter: 10 cm, height: 15 cm) with open top was used to restrict ambulation. A timer controlled by the foot pedal started computing the response latency from the moment the mouse was placed onto the cold surface. The pain-related behaviour (licking of the hind paw) was observed, and the time (seconds) of the first sign of discomfort was recorded. The cut-off time of paw lifting or licking was set at 30 s as previously reported [22].

### 2.9. Assessment of Mechanical Hyperalgesia, Paw Pressure Test

The response of the mouse against a mechanical noxious stimulus was determined using the Paw pressure test. The latency in seconds of paw withdrawal from a constant mechanical pressure (applied to its dorsal surface) was recorded. A 15-g calibrated glass cylindrical rod (diameter = 10 mm) chamfered to a conical point (diameter = 3 mm) was used to apply the mechanical force. The weight was suspended vertically between 2 rings attached to a stand and was free to move vertically. A single measure for each paw was made per animal. A cutoff time of 40 s was used [23].

### 2.10. Tissue Collection and Immunohistochemistry of the Spinal Cord

On day 15, after the behavioral measurements performed on animals belonged to the protective treatment protocol, mice were sacrificed by decapitation and the lumbar section of the spinal cord was removed, postfixed in 4% neutral buffered paraformaldehyde and cryoprotected in 30% sucrose solution at 4 °C. Slide-mounted cryostat sections (5 μm) were processed for indirect immunofluorescence histochemistry.

Five μm formalin-fixed cryostat sections were incubated for 30 min in ready-to-go blocking solution (Bio-Optica, Milan, Italy) at room temperature to block unspecific binding. The primary antibodies, incubated overnight at 4 °C, were directed against Iba1 (rabbit, 1:500; Wako Chemicals, Richmond, VA, USA) for microglial staining or against glial fibrillary acidic protein (GFAP; rabbit, 1:500; Dako, Carpinteria, CA, USA) for astrocyte staining. After rinsing in PBST, sections were incubated in donkey anti-rabbit IgG secondary antibody labelled with Alexa Fluor 488 (1:500, Invitrogen, Milan, Italy) at room temperature for 1 h. Nuclei were stained with DAPI (4′,6-diamidin-2-fenilindolo; 1:2000; Invitrogen, Milan, Italy).

Negative control sections (no exposure to the primary antisera) were processed concurrently with the other sections for all immunohistochemical studies.

Images were acquired by a motorized Leica DM6000B microscope equipped with a DFC350FX camera (Leica, Mannheim, Germany).

Quantitative analysis of GFAP and Iba1-positive fluorescence intensity was performed on at least six independent fields collected through a 40 × 0.5 NA objective for each animal.

The mean fluorescence intensity of GFAP or Iba1 was calculated by subtracting the background (multiplied by the total area) from the GFAP or Iba1 integrated intensity. The GFAP or Iba-1 signal in immunostained sections was quantified using ImageJ 1.54 software (NIH, Bethesda, MD, USA) by automatic thresholding images with the aid of the “moments” algorithm, which we found to provide the most consistent pattern recognition across all acquired images.

### 2.11. Statistical Analysis

All experiments were performed by a researcher masked to the treatment. Results were expressed as mean ± S.E.M. of 6–8 animals per group from 2 experimental replicates. To calculate the statistical significance of both behavioral and histological data, the analysis of variance was performed by one-way ANOVA. A Bonferroni significant difference procedure was used as a post hoc comparison. *p* values of less than 0.05, 0.01, or 0.001 were considered significant. Data were analyzed using the “Origin 9.1” software (OriginLab, Northampton, MA, USA).

## 3. Results

### 3.1. UHPLC-DAD-HRMS Profile of up-CS

**Figure 1:**
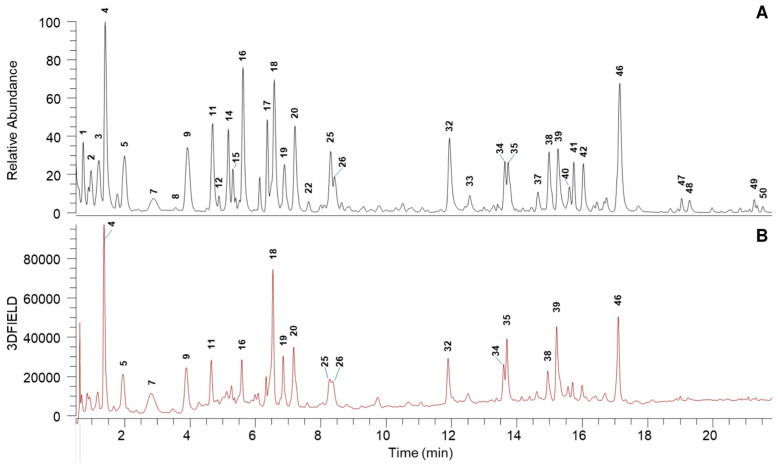
UHPLC-DAD-HRMS profile of up-CS extract: (**A**) (–)-HRMS trace (*m*/*z* 100–1000); (**B**) DAD trace (200–600 nm).

An untargeted analysis by UHPLC-HRMS/MS was performed to determine the profile of the main phytochemicals of up-CS. Metabolite assignments were carried out based on UV and HRMS data (accurate mass, isotopic distribution and HRMS/MS fragmentation pattern) along with MS data reported in the literature or databases, chemo-taxonomic data and the use of standard compounds, whenever available. The lists of identified metabolites, numbered according to elution order are reported in Table 1 and UHPLC-DAD-HRMS profile up-CS is given in Figure 1.

Up-CS displayed a very complex profile, and the untargeted analysis allowed us to identify 50 phenolic compounds, belonging mainly to two main classes: n-galloylated glucoses and flavonoids (Table 1).

Galloylated glucoses ranged from mono- to tetra-galloylated derivatives. UHPLC-DAD profile indicated that two digalloyl-O-glucose isomers (**11** and **20**), trigalloyl-O-glucose (**32**) and tetragalloyl-O-glucose (**39**), together with gallic acid (**4**), siliquapyranone (**25**) and its hydrate derivative (**19**), were the most abundant galloyl derivatives of up-CS and they are regarded as distinctive phytochemicals of carob pods [24,25,26].

Different subclasses of flavonoid compounds were detected in up-CS, mainly: flavanols, proanthocyanidin dimers and trimers, C-glycosyl flavones, dihydroflavonols and flavonol glycosides. The flavanols catechin (**18**), gallocatechin (**9**), and gallocatechin dimer (**7**), the C-glycosyl flavones of apigenin (**34** and **35**), and the flavonols myricitrin (**38**) and quercetin-3-O-dehoxyhexose (**46**) resulted the main flavonoid derivatives of the extract according to UHPLC-DAD profile, these data are in agreement with the phytochemical composition of carob pods [24,25,26]. According to the literature data, the polyphenols detected in up-CS, such as gallotannin, and flavonols, are well known for their antioxidant capacity and their impact on human health [26].

### 3.2. In Vivo Experiments

**Figure 2:**
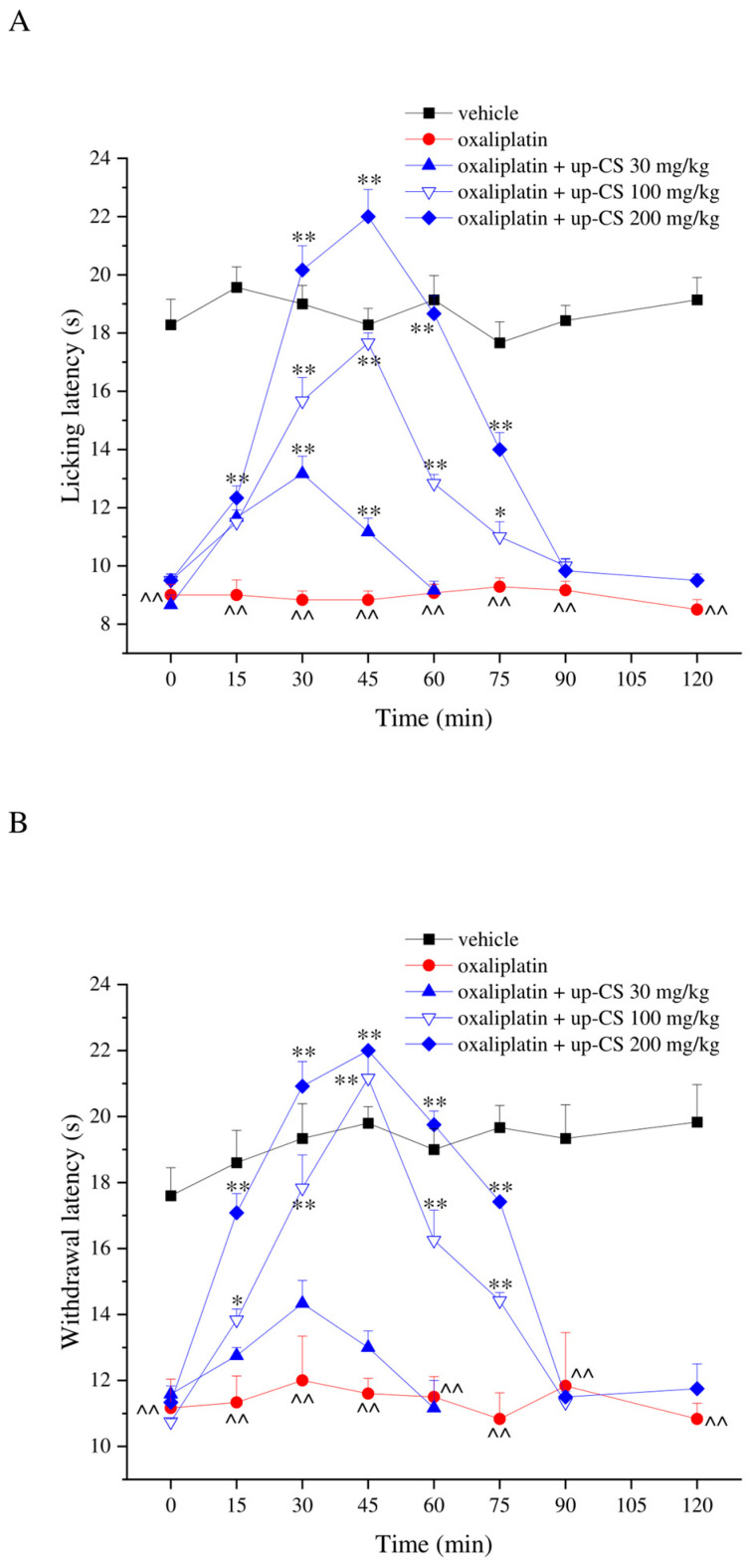
Therapeutic effect of up-CS on oxaliplatin-induced hypersensitivity. Pain threshold against (**A**) a non-noxious thermal stimulus (Cold plate test) and a (**B**) noxious mechanical stimulus (Paw pressure test) was measured. Ten oxaliplatin (2.4 mg/kg, i.p.) injections were performed over two weeks to induce hypersensitivity. On day 15, up-CS was acutely *per os* administered (30–200 mg/kg), pain measurements were performed before and 15, 30, 45, 60, 75, 90 and 120 min after treatments. Control animals received vehicles. Each value represents the mean ± S.E.M. of 6–8 animals per group performed in 2 experimental sets. Statistical analysis is one-way ANOVA followed by Bonferroni’s post hoc comparison. ^^ *p*  <  0.01 vs. vehicle treated group; * *p*  <  0.05 and ** *p*  <  0.01 vs. oxaliplatin treated group.

The acute pain-relieving profile of up-CS extract was evaluated in a mouse model of oxaliplatin-induced neuropathy, oral treatments were performed when neuropathy was well established to assess the therapeutic profile of the compound. Oxaliplatin neurotoxicity was characterized by the development of mechanical hyperalgesia and thermal allodynia after repeated administration (10 intraperitoneal injection in two weeks at the dose of 2.4 mg/kg) as shown in Figure 2. On day 15, the response of the animals challenged with a cold non-noxious stimulus was recorded. The pain threshold of oxaliplatin-treated mice decreased to 9.0 ± 0.4 s from 18.3 ± 0.9 s recorded in the vehicle group. Acute treatment with up-CS in a range of dose from 30 mg/kg to 200 mg/kg provoked a dose-dependent pain-relieving effect with a peak of efficacy at 45 min after injection. The extract at the highest dose of 200 mg/kg completely counteracted the thermal allodynia evoked by oxaliplatin-repeated treatment from 30 to 60 min after injection. The effect remained statistically significant up to 75 min (Figure 2A). The dose of 100 mg/kg showed a similar profile although with a reduced efficacy. The low dose of up-CS was still active reaching the peak of efficacy 30 min after the injection (Figure 2A). Similar results were obtained using a mechanical noxious stimulus (Paw pressure test; Figure 2B). The withdrawal latency of the posterior paws of oxaliplatin-treated mice was significantly reduced in comparison to the vehicle group, up-CS reverted oxaliplatin-induced mechanical hyperalgesia at the dose of 200 mg/kg, being active from 15 min up to 75 min. The dose of 100 mg/kg was effective from 15 to 75 min despite with a lower efficacy while the dose of 30 mg/kg was inactive (Figure 2B).

**Figure 3:**
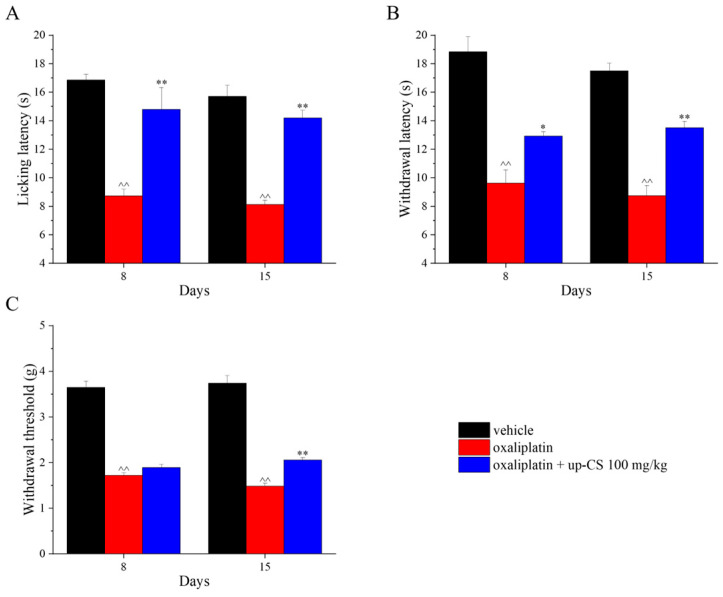
Protective effect of up-CS on oxaliplatin-induced hypersensitivity. Pain threshold against (**A**) a non-noxious thermal (Cold plate test), a (**B**) noxious mechanical (Paw pressure test) and (**C**) a non-noxious mechanical stimulus was measured. Pain measurements were performed on days 8 and 15 after the beginning of oxaliplatin and up-CS administrations, 24 h after the last treatment. Ten oxaliplatin (2.4 mg/kg, i.p.) and up-CS (100 mg/kg, *per os*) administrations were concomitantly performed over two weeks. Control animals received vehicles. Each value represents the mean ± S.E.M. of 8 animals per group performed in 2 experimental sets. Statistical analysis is one-way ANOVA followed by Bonferroni’s post hoc comparison. ^^ *p*  <  0.01 vs. vehicle treated group; * *p* < 0.05 and ** *p*  <  0.01 vs. oxaliplatin treated group.

Repeated treatments with 100 mg/kg up-CS were carried out to evaluate the protective effect of the extract in the development of oxaliplatin-induced neuropathic pain. up-CS was administered the same days of the chemotherapeutic drug (10 injections over two weeks) and the effects of the treatment on somatic pain evaluating thermal and mechanical allodynia (Cold plate and von Frey tests) and mechanical hyperalgesia (Paw pressure test) were registered on days 8 and 15. All the behavioural measurements were carried out 24 h after the last administration of oxaliplatin and/or up-CS to highlight the protective role of the extract and not its acute effect previously described in the same animal model of chronic pain. Oxaliplatin significantly reduced the mouse pain threshold in all the tests performed in comparison to the control group treated with vehicles (Figure 3). up-CS, repeatedly administered, counteracted thermal allodynia both on day 8 and 15 as demonstrated by the Cold plate test (Figure 3A). Moreover, a significant increase of the withdrawal threshold of the posterior paws was also recorded when the animals were challenged with a noxious mechanical stimulus (Paw pressure test; Figure 3B). In this test, the effect appeared to be maximum at the end of the treatment (day 15). The last measurement of somatic pain showed that up-CS siliqua was partially active in reducing mechanical allodynia only on day 15 after the beginning of the experiment (Figure 3C).

We also evaluated the emotional aspects that frequently accompany neuropathic pain conditions [27]. Anxiety and depression related disorders have been evaluated with the Elevated plus maze and Open field tests (Supplementary Figures S1 and S2, respectively). Oxaliplatin treatment protocol did not alter significantly parameters as the time spent by the animals in the center of the arena, the distance travelled in the open or in the closed arms and the time mobile (Supplementary Figure S1). Nevertheless, up-CS increased the distance travelled by the mice in the open arm in the elevated plus maze test and the time spent by the animal in the corner of the Open field arena in comparison to oxaliplatin-treated group (Supplementary Figure S2).

### 3.3. Ex Vivo Analysis of Glial Cells in the Spinal Cord

**Figure 4:**
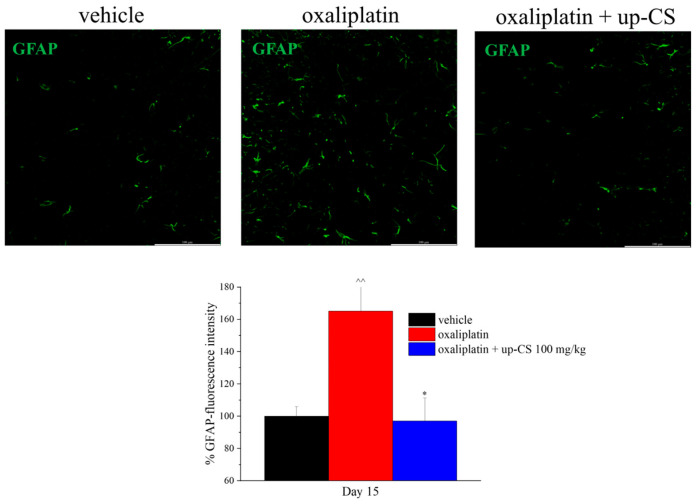
Effect of up-CS repeated treatment on astrocyte activation in the spinal cord. Immunofluorescence analysis was performed on day 15, at the end of the treatment protocol for the evaluation of the protective effect of up-CS against oxaliplatin-induced neurotoxicity. Astrocytes were studied in the dorsal horn of the lumbar spinal cord using a GFAP antibody. Histograms show the quantitative analysis of GFAP-fluorescence intensity while representative images of the dorsal horn at ×40 magnification were shown. Data were expressed as mean ± S.E.M. of 4–6 mice, performed analyzing 4 slices for each animal (2 independent field for each dorsal horn). Statistical analysis is one-way ANOVA followed by Bonferroni’s post hoc comparison. ^^ *p*  <  0.01 vs. vehicle treated group; * *p* < 0.05 vs. oxaliplatin treated group.

The capability of up-CS repeated treatment to protect the nervous system against the maladaptive plasticity evoked by a neuropathic pain state was investigated in the dorsal horn of the spinal cord. Repeated oxaliplatin injections determined astrocytes activation measured as GFAP- fluorescence intensity in comparison to the control group. The extract repeated treatment significantly prevented the astrocytic response to the chemotherapeutic drug (Figure 4). As already described in previous paper [23], microglia were not altered by oxaliplatin on day 15 in the spinal cord; the co-treatment with up-CS did not modify this condition (Supplementary Figure S3).

## 4. Discussion

This study was designed to investigate in a neuropathic pain animal model, the potential beneficial effect of the *Ceratonia siliqua* unripe pod extract (up-CS) on oxaliplatin-induced neuropathic pain, a serious side effect that strongly impairs the quality of life of cancer patients treated with oxaliplatin. A qualitative analysis of phytochemical compounds of the studied up-CS was also performed.

The *C. siliqua* variety we studied originates from Apulian Region as reported in Materials and Methods Section.

The qualitative phytochemical profile of the up-CS was evaluated by UHPLC-HRMS/MS analysis. As expected, results showed the predominance of phenolic compounds mainly represented by n-galloylated glucoses and flavonoids. Among n-galloylated glucoses, di-, tri-, and tetra-galloyl glucose were found, as well as gallic acid and siliquapyranone and its hydrate derivative. Interestingly, siliquapyranone is a tannic acid tetrahydropyran-2-one that has been only recently isolated from *C. siliqua* [28]. Among flavonoids, catechin, gallocatechin, myricitrin, quercetin 3-O-deoxyexose and *C*-glycosyl flavones of apigenin were detected.

These data agree with the phytochemical composition reported by other authors in ripe or unripe carob pods [12,14]. Several polyphenols, flavonoids, and flavonols are, in fact, commonly described.

As above reported, we investigated the ability of unripe carob pod extracts to reduce oxaliplatin-induced neuropathy in a mouse model of oxaliplatin-induced neuropathic pain. The carob extract was administered orally and classical tests to assess mechanical and thermal allodynia (i.e., by von Frey test and Cold plate test, respectively) and mechanical hyperalgesia (i.e., by Paw pressure test) induced by oxaliplatin, were used. Interestingly, the up-CS acute treatment obtained a dose-dependent pain-relieving effect and the highest dose (i.e., 200 mg/kg) was able to counteract the thermal allodynia up to 75 min. A similar result was observed with the Paw pressure test. The anti-hypersensitivity effect of up-CS is remarkable if compared with that evoked by duloxetine, a standard drug clinically used for the management of chemotherapy-induced neuropathy [29], in the same animal model. Indeed, 15 mg/kg duloxetine, acutely *per os* administered, showed a lesser and shorter effect in comparison to up-CS 200 mg/kg. Even the repeated administration of up-CS (100 mg/kg), performed concomitantly with oxaliplatin, was able to protect mice from the development of oxaliplatin-induced allodynia and hyperalgesia. As a secondary objective, we were interested in investigating the emotional aspect of neuropathic pain which is a common comorbidity of neuropathy [30]. Nevertheless, we recorded only a few differences between the control animals and the oxaliplatin-treated group. These results did not allow us to provide clear evidence of a beneficial effect of up-CS on anxiety and depression. Finally, we found that the repeated administration of up-CS prevented the astrocyte activation commonly observed as a response to the neuropathic pain induced by oxaliplatin, while microglia was not affected by oxaliplatin, at the end of the treatment protocol, as already demonstrated [23]. Consequently, no effect of up-CS was observed on microglia.

To date, the antinociceptive properties of carob pods have been poorly investigated since it has been mainly studied in gastrointestinal disorders, digestives diseases, and diabetes.

At our knowledge, there is in fact only one study in which the antinociceptive and anti-inflammatory properties of *C. siliqua* L. (i.e., in the ribe status) have been evaluated [10]. These authors established animal models of inflammation and pain by carrageenan and formalin injections, respectively. They showed that the ethanol extract of the carob leaves was able to decrease the paw licking time both in early (0–5 min) and late (15–30 min) phases after formalin injection compared with controls. They observed the same effect when the hotplate test was performed. The ethanol extract of carob leaves also caused dose dependent inhibition in paw oedema after the injection of carrageenan. The lack of effects when opioid receptors were blocked by the opioid receptor antagonist naloxone, induced the authors to suggest a central anti-nociceptive activity of carob due to a different mechanism compared with that exerted by opioids.

Overall, the phytochemical content we detected in the up-CS may explain the improvement of the oxaliplatin-induced neuropathy in our animal model. Several evidence have, in fact, shown the antinociceptive effects of many polyphenols and flavonoids we identified in the up-CS. Main examples are, for instance, polyphenols such as catechins and gallic acid, flavonoids such as myricitrin and quercetin.

In inflammatory pain animal models obtained by carrageenan and formalin administration and in a neuropathic pain animal model obtained by spinal nerve ligation, oral (-)-epicatechin was able to significantly reduce carrageenan-induced inflammation and nociception, and formalin-induced and nerve injury-induced nociception [31]. Also, intrathecal epigallotechin-3-gallate counteracted the neuropathic pain induced by spinal nerve ligation as shown by a dose-dependently increase of the paw withdrawal threshold at the spinal nerve ligation site [32]. Similarly, oral gallic acid was able to decrease the spontaneous nociception triggered by TRPA1 agonists as well as to decrease thermal and mechanical allodynia in a neuropathic pain animal model [33].

The intraperitoneal administration of myricitrin abolished nociception induced by bradykinin in mice, reduced that induced by cinnamaldehyde, reduced menthol-induced mechanical allodynia, and prevented cold allodynia induced by menthol [34].

A recent review [35] well summarizes the current knowledge on the anti-nociceptive effects of quercetin by discussing evidence obtained in several pain animal models. Despite evidence regarding the pain-relieving properties of single compounds, it is important to consider that the efficacy of a phytotherapeutic approach is based on the combined contribution of a mixture of constituents able to act with different mechanisms of action to the multi-factorial nature of neuropathy pathogenesis.

Some limitations of our study can be highlighted. We evaluated only one extract obtained by one variety of *C. siliqua* although other varieties or preparation methods are available. In particular, it has been reported that qualitative and quantitative physicochemical characteristics may vary according to the variety of the plant, the geographical area in which it is cultivated and collected, and the part of the plant that is used (e.g., pods, pulp, leaves) [14,28] with a substantial expected variability in the medicinal properties. Also, due to the exploratory nature of our study, we did not provide insights on the mechanism of action of specific detected compounds being, at this stage of our research, more interested in establishing the beneficial effect of up-CS in pain relief. Finally, to effectively translate our findings to patients with OIPN, a pharmacokinetic study should be implemented. We are aware of these limitations that will be overcome in further studies.

However, the number of studies investigating *C. siliqua* L. is quite scanty. Thus, this study adds important information on *C. siliqua* L. with a focus on its ability to ameliorate OIN.

## 5. Conclusions

Our study is the first one that evaluates the ability of the unripe carob pods in ameliorating chemotherapy-induced neuropathy. Overall, it contributes to the knowledge on *C. siliqua* L. properties by highlighting its pain relieving activity in the critical painful condition related to the administration of the neurotoxic, anticancer agent oxaliplatin. The current lack of adjuvants and/or revertants of this serious side effect highly justifies the search of potentially active synthetic, semi-synthetic, or natural compounds and our results support *C. siliqua* L. as a potential candidate.

## Figures and Tables

**Table 1:** UHPLC-HRMS/MS data of compounds detected in up-CS extract.

N	Compound	RT [min]	Molecular Formula	[M-H]^−^ (*m*/*z*)	Error (ppm)	Diagnostic Fragments	MSI Level
**1**	Galloyl-glucose	0.7	C_13_H_16_O_10_	331.0671	−0.3	211.0249, 169.0144, 125.0245	2
**2**	Galloyl-glucose	0.9	C_13_H_16_O_10_	331.0670	−0.4	169.0144, 125.0245	2
**3**	Galloyl-glucose	1.2	C_13_H_16_O_10_	331.0668	−0.8	169.0144, 125.0245	2
**4**	Gallic acid	1.4	C_7_H_6_O_5_	169.0143	0.6	125.0245	1
**5**	Digalloyl-glucose	2.0	C_20_H_20_O_14_	483.0778	−0.5	331.0675, 313.0675, 169.0144, 125.0245	2
**6**	Gallocatechin trimer	2.8	C_45_H_38_O_21_	913.1844	0.4	609.1254, 423.0724, 305.0667	2
**7**	Gallocatechin dimer	2.9	C_30_H_26_O_14_	609.1251	−0.4	423.0724, 305.0670, 125.0245	2
**8**	Galloyl-dihexoside	3.6	C_19_H_26_O_15_	493.1203	0.8	331.0674, 313.0569, 2710462, 211.0250, 169.0144, 125.0245	2
**9**	Gallocatechin	3.9	C_15_H_14_O_7_	305.0666	−0.6	179.0352, 125.0245	2
**10**	Butyryl-hexose-pentose	4.7	C_15_H_26_O_11_	427.1456 ^a^	−0.6	293.0882, 191.0563, 149.0457, 87.0452	2
**11**	Digalloyl-glucose	4.7	C_20_H_20_O_14_	483.0775	−1.2	331.0676, 313.0570, 169.0144, 125.0245	2
**12**	Galloyl-parasorboside hydrate	4.9	C_19_H_26_O_13_	461.1302	0.7	331.0674, 309.1195, 169.0144, 125.0245	2
**13**	Catechin-gallocatechin	5.2	C_30_H_26_O_13_	593.1304	−0.1	407.0778, 289.0721, 177.0195, 125.0245	2
**14**	Butyryl-hexose-pentose	5.2	C_15_H_26_O_11_	427.1451 ^a^	−1.4	293.0883, 191.0564, 149.0457, 87.0452	2
**15**	Galloyl-parasorboside	5.3	C_19_H_24_O_12_	443.1192	−0.8	331.0676, 313.0561, 211.0251, 169.0144, 125.0246	2
**16**	Methylgallate	5.6	C_8_H_8_O_5_	183.0298	−0.4	124.0167	2
**17**	Procyanidin B1	6.5	C_30_H_26_O_12_	577.1352	−0.4	407.0775, 289.0721, 177.0194, 125.0245	1
**18**	Catechin	6.6	C_15_H_14_O_6_	289.0717	−0.5	245.0821, 203.0715, 109.0296	1
**19**	Siliquapyranone hydrate	6.9	C_26_H_30_O_17_	613.1408	−0.5	461.1306, 331.0674, 169.0144, 125.0245	3
**20**	Digalloyl-glucose	7.2	C_20_H_20_O_14_	483.0775	−1.2	331.0676, 313.0570, 271.0462, 211.0250, 169.0144, 125.0245	2
**21**	Catechin trimer (Procyanidin C1)	7.2	C_45_H_38_O_18_	865.1998	1.4	577.1356, 289.0721	2
**22**	Dihydromyricetin *O*-hexose	7.6	C_21_H_22_O_13_	481.0988	−0.8	463.0891, 319.0469, 301.0358, 283.0251, 193.0144, 125.0245	2
**23**	Trigalloyl-glucose	8.1	C_27_H_24_O_18_	635.0888	−0.3	465.0680, 313.0569, 169.0144, 125.0245	2
**24**	Vanillic acid-hexoside	8.2	C_14_H_18_O_9_	329.0877	−0.2	167.0352, 123.0453	2
**25**	Siliquapyranone	8.3	C_26_H_28_O_16_	595.1297	−1.3	443.1200, 331.0675, 169.0144, 125.0245	1
**26**	Dihydromyricetin	8.4	C_15_H_12_O_8_	319.0458	−0.5	301.0359, 193.0144, 125.0245	2
**27**	Trigalloyl-glucose	8.8	C_27_H_24_O_18_	635.0888	−0.3	465.0680, 313.0569, 169.0144, 125.0245	2
**28**	Epicatechin	9.3	C_15_H_14_O_6_	289.0717	−0.5	245.0821, 203.0715, 109.0296	2
**29**	Aromadendrin *O*-hexoside	9.5	C_21_H_22_O_11_	449.1082	−1.6	287.0565, 259.0615, 125.0245, 151. 0039	2
**30**	Catechin dimer	10.0	C_30_H_26_O_12_	577.1352	−0.4	407.0775, 289.0721, 177.0194, 125.0245	2
**31**	Epigallocatechin gallate	10.8	C_22_H_18_O_11_	457.0780	0.7	305.0671, 169.0144, 125.0245	1
**32**	Trigalloyl-glucose	11.9	C_27_H_24_O_18_	635.0888	−0.3	465.0680, 313.0569, 169.0144, 125.0245	2
**33**	Gallocatechin gallate	12.6	C_22_H_18_O_11_	457.0780	0.7	305.0671, 169.0144, 125.0245	2
**34**	Isoshaftoside or Shaftoside	13.6	C_26_H_28_O_14_	563.1403	−1.0	443.0988, 473.1096, 383.07747, 353.0667	2
**35**	Isoshaftoside or Shaftoside	13.7	C_26_H_28_O_14_	563.0141	0.0	443.0988, 473.1096, 383.07747, 353.0667	2
**36**	Myricetin *O*-hexoside	13.8	C_21_H_20_O_13_	479.0836	0.9	317.0300, 316.0228, 287.0201, 271.0250, 151.0039	2
**37**	(Epi)catechin gallate	14.6	C_22_H_18_O_10_	441.0832	1.1	289.0721, 169.0144, 125.0245	2
**38**	Myricitrin	15.0	C_21_H_20_O_12_	463.0880	−0.3	317.0300, 316.0228, 287.0201, 271.0250, 151.0039	1
**39**	Tetragalloyl-glucose	15.3	C_34_H_28_O_22_	787.1006	0.8	617.0790, 465.0677, 313.0568, 169.0144, 125.0245	2
**40**	Isoquercitrin	15.6	C_21_H_20_O_12_	463.0885	0.4	300.0278, 271.0251, 255.0302, 151.0039	1
**41**	Phloretin Di-*C*-glucoside	15.7	C_27_H_34_O_15_	597.1820	−1.2	477.1409, 387.1087, 357.0981	2
**42**	Coumaroyl-galloyl-hexoside	16.0	C_22_H_22_O_12_	477.1036	−0.6	331.0678, 169.0145, 163.0403, 125.0245	2
**43**	Naringenin *O*-hexoside	16.3	C_21_H_22_O_10_	433.1144	0.9	271.0614, 151.0038, 119.0503	2
**44**	Quercetin 3-*O*-pentoside	16.4	C_20_H_18_O_11_	433.0783	1.4	300.0278, 271.0251, 151.0039	2
**45**	Quercetin 3-*O*-pentoside	16.7	C_20_H_18_O_11_	433.0778	0.4	300.0278, 271.0251, 151.0039	2
**46**	Quercetin 3-*O*-deoxyhexoside	17.2	C_21_H_20_O_11_	447.0933	−0.1	300.0278, 271.0251, 151.0039	2
**47**	Kaempferol *O*-deoxyhexoside	19.0	C_21_H_20_O_10_	431.0985	0.1	285.0407, 255.0302, 151.0037	2
**48**	Eriodictyol	19.3	C_15_H_12_O_6_	287.0565	1.1	151.0038, 135.0453	1
**49**	Naringenin	21.3	C_15_H_12_O_5_	271.0614	0.5	151.0038, 119.0503	1
**50**	Genistein	21.5	C_15_H_10_O_5_	269.0458	0.6	133.0297	1

^a^ formic acid adduct, [M-H+FA]^−^.

## Data Availability

Data supporting the findings of the study are available from the corresponding author upon a reasonable request.

## Supplementary Materials

The following supporting information can be downloaded at: https://www.mdpi.com/article/10.3390/nu17010121/s1, Figure S1: Effect of up-CS repeated treatment in the Elevated Plus Maze (EPM); Figure S2: Effect of up-CS repeated treatment in the Open Field Test (OFT); Figure S3: Effect of up-CS repeated treatment on microglia in the spinal cord.
