# Supplementary material for: The Unripe Carob Extract (Ceratonia siliqua L.) as a Potential Therapeutic Strategy to Fight Oxaliplatin-Induced Neuropathy
Source: Nutrients. 2024 Dec 30;17(1):121. doi: 10.3390/nu17010121 (PMC11723348; doi:10.3390/nu17010121)
Supplement: Supplementary file 1 [file nutrients-17-00121-s001.zip › nutrients-3372247-supplementary.pdf]

The unripe Carob extract *(Ceratonia siliqua* L.) as a potential therapeutic strategy to fight oxaliplatin-induced neuropathy

Laura Micheli[1], Marilena Muraglia[2], Filomena Corbo[2], Maria Lisa Clodoveo[3], Roberta Tardugno[2], Valentina Santoro[4,5], Anna Lisa Piccinelli[4,5], Lorenzo Di Cesare Mannelli[1], Stefania Nobili[1], Carla Ghelardini[1]

[1]Department of Neurosciences, Psychology, Drug Research and Child Health (NEUROFARBA), Section of Pharmacology and Toxicology, University of Florence, 50139 Florence, Italy.
[2]Department of Pharmacy-Drug Science, University of Bari Aldo Moro, 70125 Bari, Italy
[3]Interdisciplinary Department of Medicine, School of Medicine, University of Bari Aldo Moro, 70124 Bari, Italy.
[4]Department of Pharmacy, University of Salerno, 84084 Fisciano, Italy
[5]National Biodiversity Future Center (NBFC), 90133 Palermo, PA, Italy

*Corresponding authors: Dr. Laura Micheli, Department of Neurosciences, Psychology, Drug Research and Child Health (NEUROFARBA), Section of Pharmacology and Toxicology, University of Florence, Florence, Italy. E-mail: laura.micheli@unifi.it; Tel: +39 0552758395 and Marilena Muraglia, Department of Pharmacy-Drug Science, University of Bari Aldo Moro, 70125 Bari, Italy E-mail: marilena.muraglia@uniba.it

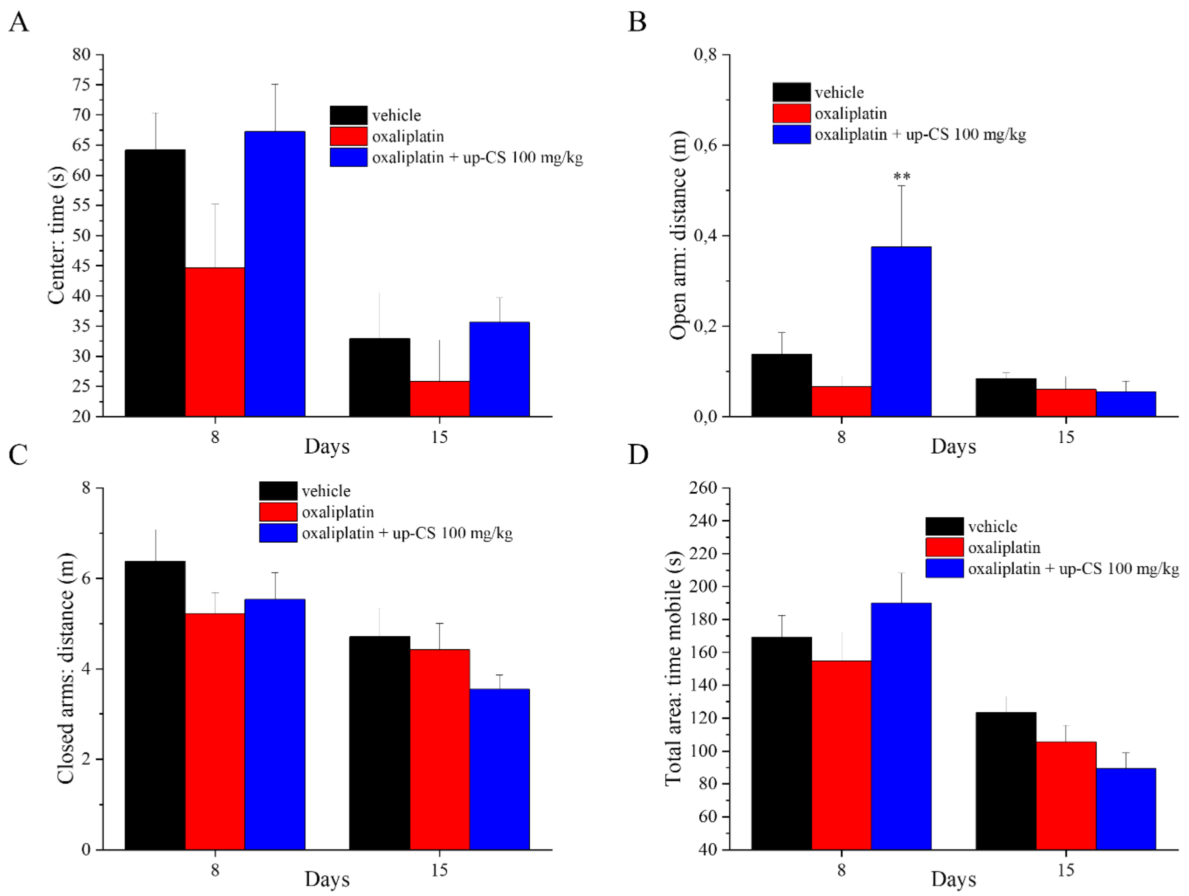

Supplementary Figure S1. Effect of up-CS repeated treatment in the Elevated Plus Maze (EPM). (A) The time spent in the center, the distance travelled in the open (B) and closed (C) arms and the (D) total time mobile were analyzed using the EPM on days 8 and 15 after the beginning of oxaliplatin and up-CS administrations, 24 h after the last treatment. Ten oxaliplatin (2.4 mg/kg, i.p.) and Ceratonia siliqua extract (100 mg/kg, per os) administrations were concomitantly performed over two weeks. Control animals were treated with vehicles. Each value represents the mean ± S.E.M. of 8 animals per group performed in 2 experimental sets. Statistical analysis is one-way ANOVA followed by Bonferroni's post hoc comparison. $^{**}P < 0.01$ *vs* oxaliplatin treated group.

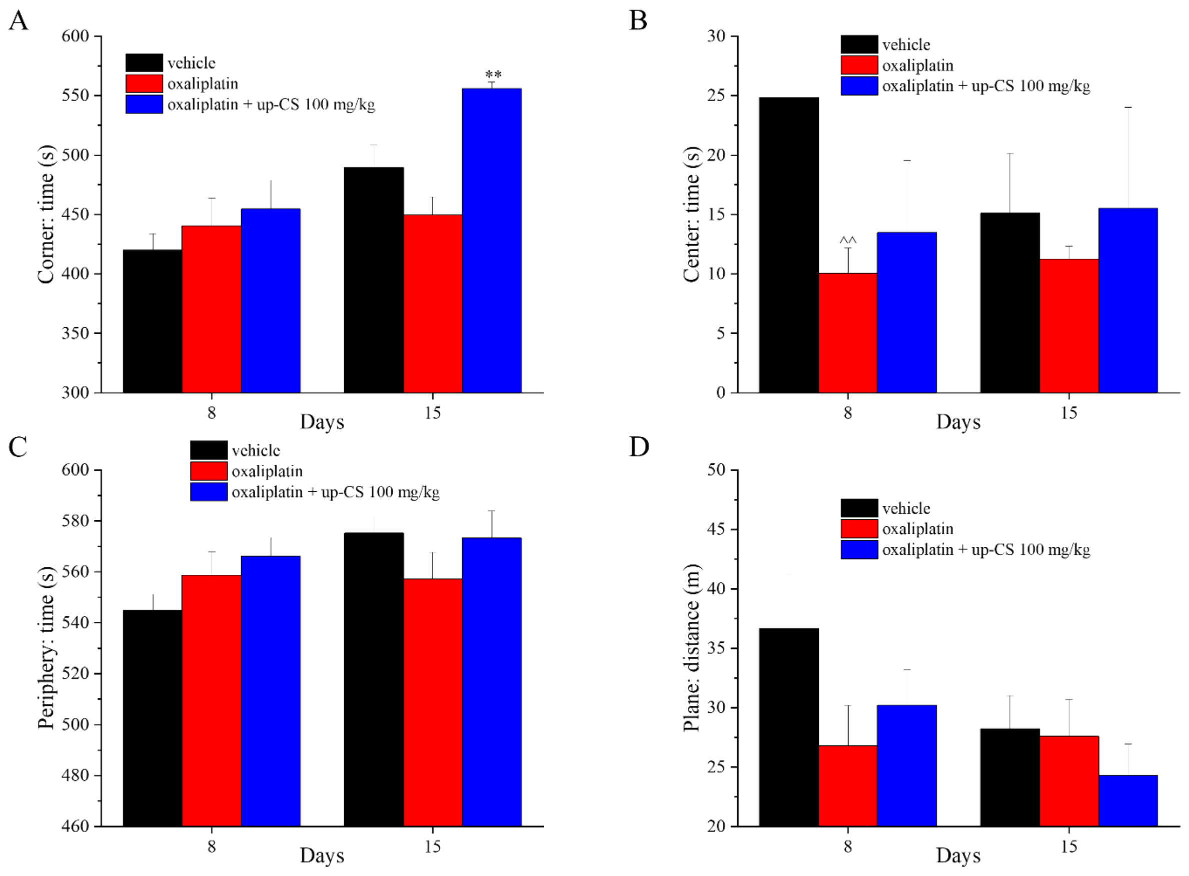

Supplementary Figure S2. Effect of up-CS repeated treatment in the Open Field Test (OFT). The time spent in the (A) corner, (B) center, (C) periphery and the (D) total distance travelled were analyzed using the OFT on days 8 and 15 after the beginning of oxaliplatin and Ceratonia siliqua extract administrations, 24 h after the last treatment. Ten oxaliplatin (2.4 mg/kg, i.p.) and up-CS (100 mg/kg, per os) administrations were concomitantly performed over two weeks. Control animals were treated with vehicles. Each value represents the mean ± S.E.M. of 8 animals per group performed in 2 experimental sets. Statistical analysis is one-way ANOVA followed by Bonferroni's post hoc comparison. ^^$P < 0.01$ *vs* vehicle treated group; **$P < 0.01$ *vs* oxaliplatin treated group.

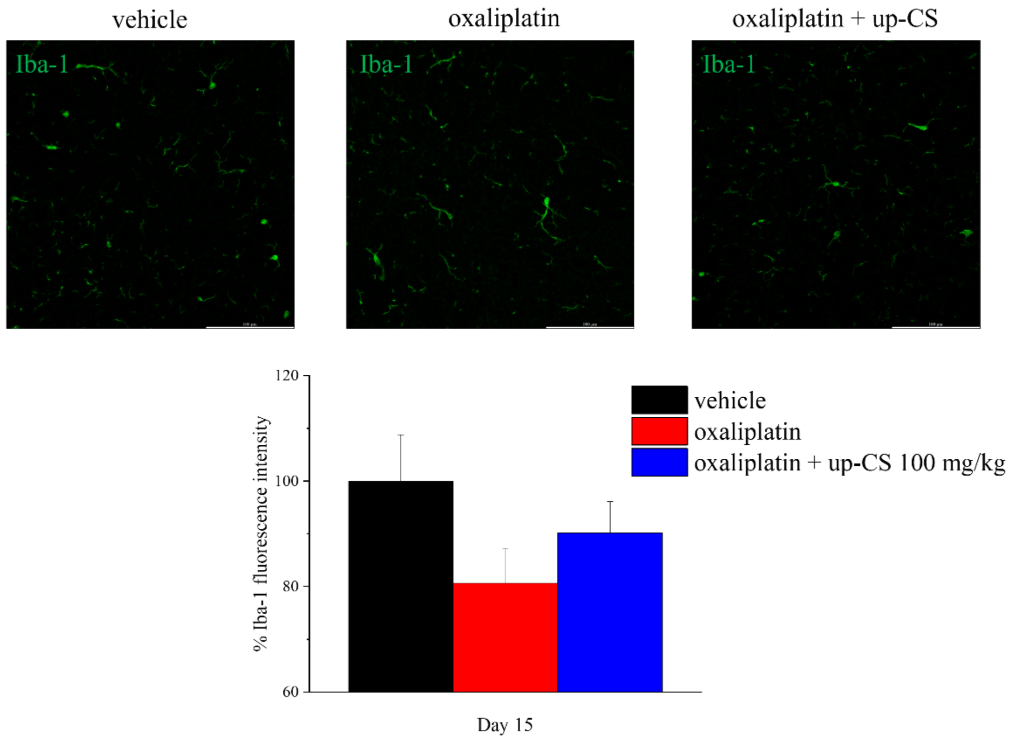

Supplementary Figure S3. Effect of up-CS repeated treatment on microglia in the spinal cord. Immunofluorescence analysis was performed on day 15, at the end of the treatment protocol for the evaluation of the protective effect of up-CS against oxaliplatin-induced neurotoxicity. Microglia was studied in the dorsal horn of the lumbar spinal cord using the Iba1 antibody. Histograms show the quantitative analysis of Iba1-fluorescence intensity while representative images of the dorsal horn at x 40 magnification were shown. Data were expressed as mean ± S.E.M. of 4-6 mice, performed analyzing 4 slices for each animal (2 independent field for each dorsal horn). Statistical analysis is one-way ANOVA followed by Bonferroni's post hoc comparison.
